# The Mobile Alliance for Maternal Action Text Message–Based mHealth Intervention for Maternal Care in South Africa: Qualitative User Study

**DOI:** 10.2196/14078

**Published:** 2020-06-29

**Authors:** Jesse Coleman, Jaran Eriksen, Vivian Black, Anna Thorson, Abigail Hatcher

**Affiliations:** 1 Wits Reproductive Health & HIV Institute School of Medicine University of Witwatersrand Johannesburg South Africa; 2 Department of Global Public Health Karolinska Institutet Stockholm Sweden; 3 Division of Clinical Pharmacology Department of Laboratory Medicine Karolinska Institutet at Karolinska University Hospital Stockholm Sweden; 4 Department of Clinical Microbiology and Infectious Diseases Faculty of Health Sciences University of the Witwatersrand Johannesburg South Africa; 5 School of Public Health Faculty of Health Sciences University of the Witwatersrand Johannesburg South Africa

**Keywords:** maternal health, text messaging, focus groups, South Africa, mHealth, reproductive health, limited resource settings, public health, prenatal care, postnatal care

## Abstract

**Background:**

Using mobile technology to support health care (mobile health [mHealth]) has been shown to improve health outcomes across a multitude of health specialties and across the world. Exploring mHealth user experiences can aid in understanding how and why an intervention was successful. The Mobile Alliance for Maternal Action (MAMA) was a free maternal mHealth SMS text messaging service that was offered to pregnant women in Johannesburg, South Africa, with the goal of improving maternal, fetal, and infant health outcomes. We conducted focus group discussions with MAMA users to learn about their experiences with the program.

**Objective:**

The aim of this qualitative study was to gather opinions of participants of the MAMA maternal mHealth service regarding health care atmosphere, intervention use, and intervention feedback.

**Methods:**

Prenatal and postnatal women (N=15) from public antenatal and postnatal care sites in central Johannesburg who were receiving free maternal health text messages (MAMA) participated in 3 focus group discussions. Predefined discussion topics included personal background, health care system experiences, MAMA program recruitment, acceptability, participant experiences, and feedback.

**Results:**

The feedback regarding experiences with the health system were comprised of a few reports of positive experiences and many more reports of negative experiences such as long wait times, understaffed facilities, and poor service. Overall acceptability for the maternal text message intervention was high. Participants reflected that the messages were timely, written clearly, and felt supportive. Participants also reported sharing messages with friends and family.

**Conclusions:**

These findings suggest that maternal mHealth interventions delivered through text messages can provide timely, relevant, useful, and supportive information to pregnant women and new mothers especially in settings where there may be mistrust of the health care system.

## Introduction

Attendance to antenatal care and postnatal follow-up care visits which provide professional maternal and infant health services during pregnancy is important for healthy maternal, neonatal, and child health outcomes [1,2]. Such visits allow health professionals to identify and treat maternal and neonatal health issues and have been found to decrease mortality and morbidity [3]. Together antenatal care, postnatal follow-up care visits, and infant vaccinations constitute the core of the maternal, neonatal, and child health continuum of care [4].

South Africa did not meet key child and maternal mortality United Nation Millennium Development Goals (goals 4 and 5) by 2015, largely due to the high prevalence of HIV [5]; however, these mortality statistics did show improvement. In 2007, at the height of the HIV epidemic, South Africa had 48.1 infant deaths per 1000 live births which halved to 23.6 per 1000 by 2013 [6]. Between 2004 and 2015, South African child (under 5 years of age) mortality decreased from 66.9 to 38.0 deaths per 1000 [6], and in 2013, the maternal mortality ratio was estimated to be 148 per 100 000 [7]. Despite these positive changes, more improvement is needed in order to achieve national and global maternal, neonatal, and child health goals.

Mobile technology, when used to support health care services, is often referred to as mHealth (mobile health) [8]. Previous systematic reviews of maternal mHealth interventions in low- or middle-income countries have highlighted the improvement in maternal and neonatal health outcomes, but have also recommended further research into the factors contributing to successful and unsuccessful mHealth interventions in practice [9-12]. A gap in research exists because most mHealth evaluations tend to use quantitative methodology. There is a need for rigorous and continued evaluation of mHealth interventions in order to understand how they worked and why they succeeded (or did not succeed) and to ensure future mHealth interventions are implemented successfully. Process evaluations of mHealth interventions have been used only a handful of times globally [13-15] and were used as part of pilot projects to identify participant need and interest rather than to look at large-scale implementation [16-18]. A qualitative analysis [19] of a nationally implemented (in South Africa) maternal mHealth intervention included only one clinic in the study. Another South African study [20] investigated the acceptance of ad hoc use of mobile technology by patients and providers, rather than that of a specific mHealth intervention. This study aims to address gaps in knowledge by exploring the experiences of participants of the Mobile Alliance for Maternal Action South Africa (MAMA) project, an SMS text message–based maternal mHealth intervention that was offered in Johannesburg between 2012 and 2014.

### MAMA Intervention Overview

The MAMA intervention sent maternal health and infant care information by SMS text message to approximately 12,000 pregnant women and new mothers in Johannesburg, South Africa, throughout pregnancy and until their infant was one year of age [21]. At the time of recruitment, women were given the option to receive one of two types of text message content—general maternal health information or prevention of mother-to-child transmission of HIV maternal health information; however, due to a high rate of women who were pregnant and HIV-positive [22], both streams of messages contained some HIV content, such as regular HIV-testing reminders (see Multimedia Appendix 1). The difference between the prevention of mother-to-child transmission of HIV content stream and the general maternal health content stream was that approximately 20 general maternal health support–related messages were replaced with prevention of mother-to-child transmission of HIV–related messages. The intervention predated, however retrospectively, was in line with the World Health Organization Classification of Digital Health Interventions [23], whereby specific digital health interventions can be used to address health system challenges; in the case of the MAMA intervention, targeted health information was transmitted to a certain demographic (pregnancy) clients to provide health education and to decrease attrition rates [23].

Through routine operational research [22], MAMA SMS text message recipients had previously provided feedback regarding a number of contextual factors such as poverty, violence, alcohol, social support, the underresourced health care system, the high rate of HIV infection, and the high rate of miscarriage. Participant discussions of poverty and violence subthemes included topics of long-term unemployment and sharing living space with multiple other families, as well as perspectives on the effects of excessive alcohol use on both themselves and their community. Income-related concerns that were provided during feedback included not being able to afford high-quality medical services, witnessing verbal or physical fights between couples on the subject of finances, and being unable to regularly afford meals that included meat. Social support subthemes included social norms such as being able to turn to siblings and older generation members of the family for support after delivery to enable a safe and supportive environment for themselves and their newborns.

Previous nonrandomized quantitative studies [24,25] that investigated MAMA health outcomes looked at mother-infant pairs who received the MAMA SMS text messages compared to the mother-infant pairs who did not receive MAMA SMS text messages and showed that those in the intervention arm had a higher rate of antenatal care attendance, an increased likelihood of a vaginal birth, a reduced likelihood of emergency cesarean delivery, and were more likely to have attended all recommended postnatal follow-up care visits up to one year after birth. Furthermore, an analysis [26] found that the MAMA text message intervention would be a cost-effective strategy to improve antenatal care attendance and vaccination rates, even if only brought to scale in Gauteng, one of South Africa’s provinces.

## Methods

### Study Design

This was a qualitative study with an inductive and descriptive design. An inductive approach involves drawing codes, categories, or themes directly from the data, and is useful when knowledge about a phenomenon is limited [27].

### Study Setting and Participants

In late 2013 and early 2014, adult women (18 years of age or older) attending routine antenatal and postnatal follow-up care services at 3 sites were invited to participate in focus group discussions. Participants were purposively selected to identify women who were either at various stages of pregnancy or after delivery with infants less than one year of age on the day of recruitment. Potential participants were women who were already receiving MAMA messages and who were identified by asking; if the women responded affirmatively, they were invited to participate in the study. Women who agreed to participate in the study provided informed consent. Of the 21 women who were invited to participate, 15 women agreed to participate in the focus group discussions.

All three sites in the study were public health care facilities in Hillbrow, Johannesburg. Hillbrow has a high population density with high diversity, predominantly low-income households, and had an unemployment rate estimated at 23% in 2013 as well as high rates of behaviors such as alcohol use and gender-based violence [28]. In Hillbrow in 2013, 27% of women who were pregnant were HIV-positive [24].

### Study Procedures

A focus group discussion guide was created prior to the study and was designed to elicit feedback from participants about their experiences related to the intervention text messages (Multimedia Appendix 2). The topics covered general questions about the message content, usefulness, the signup procedure, and sharing of the messages with others. Focus group discussions were timed so that participants had received at least four months of messages which allowed them to have sufficient experience to provide feedback but was early enough in the intervention life span to allow for optimization, change, and improvement, if necessary.

Focus group discussions were held in a private room in an antenatal and postnatal follow-up care site that offered the intervention. A total of 3 focus group discussions, each with 4-6 participants, were held. Each discussion group lasted between 60 and 90 minutes and was conducted in English. At each focus group discussion, 2 to 4 research staff were present, one of whom was experienced in qualitative research and who acted as the moderator. The other research staff observed and translated between local languages and English, when necessary.

### Data Collection and Analysis

Audio recordings of each focus group discussion were transcribed verbatim, and managed in Dedoose [29], an online qualitative data analysis tool. Focus group discussions were separately read and coded by 3 members of the study team who then agreed on a hierarchical coding system. The hierarchical coding system was refined using an inductive-deductive approach based upon the focus group discussion interview guide and initial review of the transcripts. General categories were identified, reviewed, and then organized into major categories and subcategories to capture specific detail (Table 1). The 3 researchers then analyzed the text using latent content analysis inspired by Graneheim and Lundman [30]. When differences of opinion arose, coding was compared, reviewed, and discussed until there was consensus.

**Table 1:** Focus group discussion themes (categories and subcategories).

Category	Subcategory
Factors contextualizing the intervention	Poverty/employment/income
	Social support
	Experiences of the public health care system
	Positive
	Negative
Factors contributing to intervention success	Recruitment was facilitated by helpful staff members
	Privacy concerns were allayed
	Communication preferences
	Messages arrived regularly
	Text message content was accessible
Project feedback	Relevance of text message content
	Trust in the content
	Acceptability of the intervention

### Ethics

The study was approved by the Human Research Ethics Committee (Medical; M120649) at the University of the Witwatersrand in Johannesburg. Participation in the study was voluntary and informed consent was given by each participant prior to the collection of any personal information. Participants were informed that they were not required to disclose their HIV status.

## Results

### Participants

The 15 participants ranged in age from 20 to 36 years (median 31, IQR 7). All women were black, African, and residents of Hillbrow, Johannesburg. Women who were pregnant (prenatal, n=8) ranged from being between 26 and 39 weeks pregnant at the time of their focus group discussion, the other women (postnatal, n=7) had given birth between one week and 52 weeks prior. Each focus group discussion included both post and prenatal participants as well as a women who ranged in age from their twenties to thirties (group 1: 20-36; group 2: 28-35; group 3: 21-36 years of age). All participants received MAMA SMS text messages sent twice a week for at least 16 weeks (ie, at least 32 SMS text messages).

**Table 2:** Overview of focus group participant characteristics.

Focus group	Participants, n	Age (years), range	Prenatal, n (weeks gestation)	Postnatal, n (weeks since)
Group 1	5	20-36	3 (30-34)	2 (4; 20)
Group 2	4	28-35	2 (26; 39)	2 (1; 52)
Group 3	6	21-36	4 (34-39)	2 (17; 34)

### Experiences of the Public Health Care System

There were divergent opinions about the health care system. A few participants had positive feelings and experiences, but most expressed negative feelings. Those with positive experiences mentioned having trust in the medical procedures and the experts who work there. On the other hand, a number of participants described having poor opinions of both the health care system and staff. One participant was able to differentiate her opinion between the system and individuals who worked within it. Specific topics are explored in more detail below.

Feedback about the health care system related to HIV testing, care, and treatment was positive. There were comments about public HIV clinics being more trustworthy than private clinics that conducted HIV tests:

Sometimes the tests from the [private] doctors they come wrong but normally at the clinic, if you know you are testing at the clinic I don’t think your status would ever come wrong, if it's negative it will come back negative.

Postnatal woman, 32 years of age

In addition, there was a perceived benefit in the antenatal HIV testing services:

For me, I think the most important reason that you should book at the clinic is so that you may know your [HIV] status before you proceed with the pregnancy [and] so that your baby will be checked so that you can proceed with your pregnancy [knowing your baby is healthy].

Postnatal woman, 32 years of age

Notwithstanding HIV testing, care, and treatment services, health care–related feedback was less positive and participants were vocal about their negative health care experiences. Analyzing these experiences, 3 main barriers were identified: long wait times, poor treatment (by staff), and that staff seen to be overworked. One spoke about staff treating patients with disdain and disrespect, making them feel that they must “obey” and that they had made a mistake by becoming pregnant:

I wouldn’t suggest anyone to go to the clinic especially [clinic name] if she’s pregnant, no I wouldn’t suggest [it].... There [at the antenatal clinic] if you are pregnant you are being treated like you are stupid and if you don’t obey that stupidity they won’t help you, you don’t get the dignity as a human being… You’re just nothing just because you are pregnant which is not fair.

Prenatal woman, 33 years of age

Others described the long wait times and the perception that staff chose not to treat all patients who arrived on a given day:

People are coming 3 o’clock [in the morning]; imagine a pregnant person coming 3 o’clock to [wait until] 07:30. People have to come that early because [the staff] only see a small number [of patients a day] or you get turned away; no they shouldn’t do that.

Unidentified participant, focus group 3

Participants in focus group discussions recounted feeling scared of the treatment by staff, but explained that they continued to seek these services because of the importance of antenatal care for infant health:

The problem is that even if you try to talk to them they will tell you that you challenging them...we scared but we just thinking of our babies...I always tell my friends just go there don’t worry about how they treating us think about our babies.

Postnatal woman, 36 years of age

### Factors Contributing to Intervention Success

This section identifies aspects of the intervention that were mentioned by focus group discussion participants as being useful or enabling its use and integrating the information that they received into action. These included using a personalized and private recruitment technique, using an accepted and reliable communication method, and receiving simple, relevant, and supportive message content.

Recruitment to the text message intervention was done with the support of study staff who identified themselves as working with the study as opposed to identifying themselves as working for the healthcare facility:

I think the approach was good because it was professional she was able to explain what is it that I’m expecting and the messages which will, the usefulness of the message and what will it be helping me with so I think she was professional enough.

Prenatal woman, 36 years of age

Participants also noted that privacy was an important issue. One participant appreciated the careful way that staff invited her to take part in the mHealth intervention, using a discreet conversation that did not disclose her HIV status to others:

What I was happy for is she asked me [if I wanted to receive HIV-related messages] in private she didn’t just ask me in front of people so I just tell her that I know my status so she say those messages will help me so that I won’t be having stress or to think too much about it so every Monday or Thursday I was always waiting for the messages so that if I’m not OK if I haven’t gotten those messages I will feel better than before.

Postnatal woman, 28 years of age

### Intervention Communication

The focus group discussion included a query about preferred communication methods which aimed to identify if text messages were a barrier in any way. The discussion covered text messages, radio, email, television, and print, but did not include smartphones due to their low utilization. Participants reported that text messages were their favorite method of communication as it felt more personal, was inexpensive (“free to receive!”), ubiquitous, and easy to use.

I also think like the cell phone is the easiest way because everyone is using a cell phone ...I think the cell phone SMS is the best.

Postnatal woman, 28 years of age

Most participants in focus group discussions were able to recall that the messages were sent twice a week, with all but one participant able to recite the precise time and days of the week.

Interviewer: *So how often do you receive these SMS [messages]?*

Group response: *Twice a week, Monday and Thursday, 9 o’clock exactly.*

Being able to count on the text messages to arrive on time, all the time, was mentioned by multiple participants. Two female participants in different focus group discussions mentioned that they waited for their Monday morning message before they decided to bring their infant to the clinic, to see if that day’s message dealt with an issue that they had had over the weekend.

In South Africa, with 11 official languages, accessibility of the text message language was a concern among the implementers and was brought up at each focus group discussion. All focus group discussion participants claimed that English-language messages were not a barrier, but rather that English was the best option since it was easy to share with others who might not speak their mother tongue.

### Intervention Feedback

Feedback regarding the intervention highlighted that it provided helpful, relevant information at appropriate times, was trustworthy, and was accepted.

Message content was brought up repeatedly throughout the focus group discussions as being an enabler of trust in the intervention and source of the messages. This trust is highlighted by discussions of the timeliness of messages and relevant to issues they were dealing with.

At a time my baby had a problem with her skin I received an SMS saying you can take that aqueous cream and just rub your baby and I did that I saw the skin of my baby change and I was so happy...I was so happy thank you so much...I am getting so much advice... Thank you.

Postnatal woman, 20 years of age

Other topics that were remembered by participants included nutrition during pregnancy, how to handle being pregnant and HIV-positive, learning about and preparing for delivery, understanding how to connect with a newborn, and dealing with teething. Participants also mentioned feeling more confident in caring for their infants as a result of the messages, and believed that the messages were trustworthy and were considered expert material.

Some participants identified their mothers as barriers to having healthy children. Two varying methods of dealing with this disconnect were shared; the first was explicitly telling their mother that the messages should be followed because it came from professionals and the second was telling their mother that their (the mother’s) advice would be followed, but actually adhering to the message-based advice. The difference between which method was used was, in general, related to where their mothers were located. Women whose mothers were close by were more direct while women whose mothers were outside Johannesburg frequently used the second approach.

All participants showed an interest in continuing to receive SMS text messages after their baby reached one year of age. This was a recurring theme in all focus group discussion and was further evidenced by some focus group discussion participants claiming a willingness to pay for the messages, if necessary:

As now that I’ve received the SMS [text messages] I know about the SMS [text messages] already… if I have to pay I would pay because I know they are worthy, and SMS rate I would mind paying it.

Prenatal woman, 33 years of age

None of the focus group discussions brought up any negative comments about MAMA, nor did they have suggestions for improvement when probed for this.

## Discussion

### Principal Findings

In this qualitative study of user experiences of the MAMA maternal mHealth intervention, we found that, in contrast to poor experiences with the health system, MAMA maternal mHealth messages were considered to be reliable and useful. Despite mixed feelings regarding the quality of care provided by the health care system, participants were happy with the mHealth intervention and the content of the text messages. The text message intervention had an easy and discreet signup process, the use of text messages for communication was appropriate, and the content was accessible by the participants. The message content was reported to be relevant, trusted by the participants, and accepted by them.

Participants identified various barriers when trying to receive maternal health care and support. Each of these barriers may have been an indication of an underresourced health care system with high demand on staff. Participant feedback highlighted current health system barriers and disincentives for patients deciding how and when to attend health care facilities. Negative experiences at health care facilities, such as disrespectful staff and distrust of medical advice, among others, highlighted the need for a service that provides genuine, respectful, and trustworthy messaging for patients.

Focus group discussion participants generally had positive feedback and experiences of the SMS intervention because of the private recruitment, simple signup, and easy to understand messages that were relevant and timely. Most of the recruitment team were previously employed as HIV counselors which enabled them to be sensitive to the stigma that is related to HIV issues. This previous experience encouraged them to find recruitment methods that provided full patient confidentiality. Participants also reported having limited access to expert maternal health information outside of the intervention. Health care workers were seen as overworked and to only be able to provide limited support during maternal health care (antenatal care and postnatal follow-up care) visits. Thus, receiving timely and trustworthy maternal health information at no additional cost was seen as an enabler of good health, and it has, in fact, been shown to lead to better health [24]. Furthermore, given the national emphasis on HIV care, treatment, and support throughout South Africa over the last decade, the finding of a strong level of trust in the non-MAMA services provided by the public health care system was reassuring.

### Comparison With Previous Work

Being outside the health care system enabled study staff to differentiate themselves from the health care system and its negative connotations. Additionally, the staff had the ability to focus entirely on the patient and provide as much information and support as was necessary to ensure the patient felt comfortable with the signup process. The combination of high HIV rates in the target population, significant HIV-associated stigma, and cramped waiting rooms where recruitment took place meant that recruiters had to be tactful. The depth of trust, relevance, and acceptability reported by participants was in line with another maternal mHealth study [15] that took place in rural northern Canada and which used focus group discussions and showed patient perception of the mHealth intervention to be highly acceptable and relevant. The same study [15] also found a high level of trust in the messaging that was provided; participants mentioned that they could believe the messages because of the source. This similarity shows the perceived pedigree of the message content is an important factor with regard to both the trust and the acceptability of an mHealth intervention.

The request for additional messages and the willingness to pay suggested that the text messages were not just accepted by focus group discussion participants but welcomed. Willingness to pay for mHealth services has not been studied extensively. While a willingness to pay was identified in this study, we feel this claim should be taken in context given the previously identified poverty-related issues. Participant acceptability of the intervention might be due to lack of stigma around pregnancy and infant care, and the relatively young age of participants, who were, by definition, of childbearing age. Watkins et al [20] suggested that acceptability of mHealth interventions could be based on age; older individuals reported having difficulty reading text on their phones and were less receptive to technology-based interventions. Additionally, maternal health is an area that has virtually no stigma associated with it, unlike other health conditions such as HIV. A qualitative study in Kenya [14] that looked at the acceptability of SMS text message–based HIV support reported that many individuals had concerns about the use of HIV-related terms and highlighted the potential for accidental disclosure of their HIV status. In contrast, MAMA was designed to support maternal and infant health. This might help reduce stigma, even though discussions of HIV were held privately, and this in turn may encourage women to get care for this critical health issue.

Participant feedback suggested that mHealth interventions may feel more compassionate than in-person visits. Lester et al [30] highlighted this same issue in their study which showed HIV-positive individuals who received SMS text messages from health care workers had improved clinical health outcomes compared to that of nonrecipients; many who received the messages reported that it seemed “like someone cares” (p 1843). Patient feelings that someone cares about them and their pregnancy could be a contributing factor to the positive effect demonstrated in previous MAMA research [24,25].

### Limitations

This qualitative study included the feedback of only 15 participants in Johannesburg, South Africa, and might not be representative of the population as a whole. Additionally, there was potential for participants to be affected by social-desirability bias [32] as the focus group discussions were conducted close to the recruitment site. This could be a reason for the few critical statements about the intervention. Conversely, previous qualitative maternal mHealth studies [33,34] have shown that individuals tend to see mHealth based interventions in a positive light even before they are offered, which could partially explain the responses of participants in the current study. We are also aware that the thematic focus group discussion guide contains several closed questions which could have made the discussion less free. Surprisingly, there were no negative comments regarding MAMA. This could be due to the participants perceiving the researchers as coming from MAMA, and therefore, not wanting to give any negative feedback; however, the participants were outspoken and seemed honest when discussing other topics. We believe the participants genuinely saw MAMA as an important tool that helped them during their pregnancy. Lastly, these results should not replace acceptability testing in other situations or among other populations.

### Conclusions

Maternal mHealth interventions, delivered through text messages can provide timely, relevant, useful, and supportive information to pregnant women and new mothers, especially where mistrust in the health care system may exist. Maternal, neonatal, and child health is a field where this combination (timely, relevant, and supportive) is especially important and mHealth could be a tool used to attain maternal, neonatal, and child health goals, globally.

## Appendix

Multimedia Appendix 1 Examples of MAMA SMS.

Multimedia Appendix 2 Focus Group Discussion Guide.

## Abbreviations

MAMA: Mobile Alliance for Maternal Action
mHealth: mobile health
