# Supplementary material for: The Mobile Alliance for Maternal Action Text Message–Based mHealth Intervention for Maternal Care in South Africa: Qualitative User Study
Source: JMIR Hum Factors. 2020 Jun 29;7(2):e14078. doi: 10.2196/14078 (PMC7367535; doi:10.2196/14078)
Supplement: Multimedia Appendix 1 [file humanfactors_v7i2e14078_app1.docx]

**Appendix 2:** Examples of the MAMA SMS

| **Timing** | **Message content** |
| --- | --- |
| Week 23 of pregnancy | You need 4 clinic visits in pregnancy. A visit may take all day, so get a letter from the clinic for your work. (Doesn't have to say pregnancy.) |
| Week 32 of pregnancy | Going to your clinic appointments is important for you & your baby. Even if you feel healthy, please go. Remember you have a right to ask questions! |
| Week 35 of pregnancy | Breastmilk is the best food for your baby. It should be his very first food as soon as he's born. Your milk helps protect him from infections. |
| Week 5 postnatal | You should have a Road to Health card for your baby. Keep it safe and take it whenever you take your baby to a clinic, even if you've moved. |
| Week 6 postnatal | Vaccines help prevent your baby catching diseases. Each vaccine needs to be given at the right time. The first ones are due at 6 weeks. |
| Week 11 postnatal - HIV message | If your baby tested HIV+ at the 6-week test, make sure you get him to a clinic for treatment. If treated early, he will do very well. |
