# Supplementary material for: The Mobile Alliance for Maternal Action Text Message–Based mHealth Intervention for Maternal Care in South Africa: Qualitative User Study
Source: JMIR Hum Factors. 2020 Jun 29;7(2):e14078. doi: 10.2196/14078 (PMC7367535; doi:10.2196/14078)
Supplement: Multimedia Appendix 2 [file humanfactors_v7i2e14078_app2.docx]

**Appendix I: Focus Group Discussion Guide**

Interviewer: “Please remember that you can ask for the tape recorder to be switched off at any time in this discussion. You are all here because you are part of the [intervention], the [text messages]. So I would like to go around the room and ask everyone to introduce them.”

GENERAL

- Do you understand the goal of [the intervention]?
- Given what you have seen so far do you think [the intervention] is going to work well, or do you think that there are things they need to change to make it more useful or user-friendly to moms? What?
- Were you able to get the information and read it without any problems?
- Do you feel you can trust the [text message] information and rely on it?
- Do you find the information to be important to you?
- Are the text messages useful to you and why or why not?
- Is the information written in a way that you understand, or do you felt it is complicated?
- Do you think that the information should be simplified or is it easy to understand?
- Is there anything that you feel is inappropriate or disrespectful to you? Does anything you have received, for example, clash with your cultural beliefs?
- The inclusion of the HIV information, does that make anybody feel uncomfortable or feel it is unnecessary?
- Are you aware that you can opt out of the service at any time? How do you feel about the process of opting out?

SMS Messages

- Were you able to follow the signup process?
- How was the whole process of signing up for you? (do you manage to sign up by yourself, did someone talk you through it or help you?)
- Did any of you see the promotional pamphlet? What did you think of it?
- How much airtime do you think you used to sign up for the [text messages]?
- When you sign up, you are asked the question about HIV messages, do you understand what you are being asked?
- How often did you receive the [text messages]? Would you like more or fewer [text messages] and why?
- Did you have any negative feelings towards the [text messages]?
- Did you show the messages to anyone in your household or your partner? How did they feel about the [text messages]?’
- Have you changed the way you do things because of the information you have received through these [text messages]?
- Have you ever shared the [text messages] with anybody? If so, who was it and why?
- Has anyone saved the [text messages] and maybe went back to read them again?
- Would you like your [text messages] to come in any other language other than English?
- Did you understand the words in the [text messages]? Were any difficult to understand (give example if possible)?
- How did you feel about the way the messages spoke to you? Did they make you feel good or bad?

CONCLUSION

- Are there any questions or concerns or suggestions or comments that you have in mind concerning this whole system?

Interviewer: “Thank you very much for participating in this session. We really value your opinion. We have officially come to the end of everything.”
